# Long−term health outcome and quality of life in children with multisystem inflammatory syndrome: findings from multidisciplinary follow−up at an Italian tertiary−care paediatric hospital

**DOI:** 10.1007/s00431-024-05706-0

**Published:** 2024-09-10

**Authors:** Enza D’Auria, Stefania Maria Bova, Andrea Riccardo Dallapiccola, Raffaella De Santis, Alessandro Leone, Valeria Calcaterra, Savina Mannarino, Massimo Garbin, Sara Olivotto, Salvatore Zirpoli, Michele Ghezzi, Alice Marianna Munari, Elvira Verduci, Andrea Farolfi, Alessandra Bosetti, Veronica Perico, Pietro Capetti, Arianna Gadda, Laura Gianolio, Germana Lo Monaco, Luisa Lonoce, Roberto Previtali, Ludovica Serafini, Silvia Taranto, Pierangelo Veggiotti, Gianvincenzo Zuccotti

**Affiliations:** 1 Department of Paediatrics, Buzzi Children’s Hospital, ASST-FBF-Sacco, Via Castelvetro 32, Milan, Italy; 2 Paediatric Neurology Unit, Buzzi Children’s Hospital, Milan, Italy; 3 Department of Biomedical and Clinical Science, University of Milan, Milan, Italy; 4 International Center for the Assessment of Nutritional Status and the Development of Dietary Intervention Strategies (ICANS-DIS), Department of Food, Environmental and Nutritional Sciences (DeFENS), University of Milan, 20133 Milan, Italy; 5 Clinical Nutrition Unit, Department of Endocrine and Metabolic Medicine, IRCCS Istituto Auxologico Italiano, Milan, Italy; 6 Paediatric and Adolescent Unit, Department of Internal Medicine and Therapeutics, University of Pavia, Pavia, Italy; 7 Paediatric Cardiology Unit, Buzzi Children’s Hospital, Milan, Italy; 8 Department of Pediatric Radiology and Neuroradiology, Buzzi Children’s Hospital, Milan, Italy

**Keywords:** SARS-CoV-2, Multisystem Inflammatory Syndrome in Children (MIS-C), Long-term follow up, Glycometabolic dysfunctions, Psychosocial well-being, Quality of life

## Abstract

Multisystem inflammatory syndrome is a severe complication of SARS-CoV-2 infection in children (MIS-C). To date, data on long-term sequelae mainly concern cardiac outcomes. All ≤ 18 year olds consecutively admitted to the Buzzi Children’s Hospital with a diagnosis of MIS-C between October 1, 2020, and May 31, 2022, were followed up for up to 12 months by a dedicated multidisciplinary team. They underwent laboratory tests, multi-organ clinical and instrumental assessments, and psychosocial evaluation. 56/62 patients, 40 M, mean age 8.7 years (95% CI 7.7, 9.7), completed the follow-up. Cardiological, gastroenterological, pneumological, and neurological evaluations, including IQ and EEG, were normal. Alterations of HOMA-IR index and/or TyG index, observed in almost all patients during hospitalisation, persisted in about a third of the population at 12 months. At 6 and 12 months respectively, impairment of adaptive functions was observed in 38/56 patients (67.9%) and 25/56 (44.6%), emotional and behavioural problems in 10/56 (17.9%) and 9/56 (16.1%), and decline in QoL in 14/56 (25.0%) and 9/56 (16.1%). Psychosocial well-being impairment was significantly more frequent in the subgroup with persistent glycometabolic dysfunction at 12 months (75% vs. 40.9% *p* < 0.001).

*Conlusion: *The mechanisms that might explain the long-term persistence of both metabolic alterations and neuro-behavioural outcomes and their possible relationship are far from being clarified. Our study points out to the potential long-term effects of pandemics and to the importance of a multidisciplinary follow-up to detect potential negative sequelae in different areas of health, both physical and psychosocial.

**Table:** 

**What is known:** • *Multisystem* inflammatory syndrome in children (MIS-C) is a severe complication of SARS-CoV-2 infection. • Few data exist on the medium- and long-term outcomes of MIS-C, mostly focused on cardiac involvement. Emerging evidence shows neurological and psychological sequelae at mid- and long-term follow-up.
**What is new:** • This study reveals that MIS-C may lead to long-term glycometabolic dysfunctions joined to impairment in the realm of general well-being and decline in quality of life, in a subgroup of children. • This study highlights the importance of a long-term multidisciplinary follow-up of children hospitalised with MIS-C, in order to detect the potential long-term sequelae in different areas of health, both physical and psychosocial well-being.

**Supplementary Information:**

The online version contains supplementary material available at 10.1007/s00431-024-05706-0.

## Introduction

Multisystem inflammatory syndrome is a severe complication of SARS-CoV-2 infection in children (MIS-C) [1, 2]. According to the Centers for Disease Control and Prevention (CDC) definition [3], it is characterised by fever and laboratory evidence of inflammation with the involvement of two or more organs and systems, in the presence of a positive SARS-CoV-2 test (PCR, antigen or serology), and requires hospitalisation.

The acute phase of MIS-C has been well described [4], and most studies have also assessed its cardiac outcomes [5–7]. Conversely, data on medium- and long-term non-cardiac outcomes remain scarce [8–12], and multidisciplinary follow-up care is rare [13].

Overall, at 12 months post-admission, nearly all patients show normalisation of inflammatory markers and of cardiac injury biomarkers, as well as recovery of cardiac abnormalities [10–14].

In a retrospective study of 46 patients followed up at 6 months, Penner et al. reported neurological abnormalities in 39% of patients, and emotional difficulties in 20%, in the context of normalisation of laboratory markers of inflammation and renal parameters, and disappearance of ENT signs (dysphonia, anosmia, dysgeusia, dysphagia) [13]. Otten et al. conducted a longitudinal study in 29 children admitted to the ICU with MIS-C. At 6 months of follow-up, this sample showed normal intelligence, but more emotional and behavioural problems, a higher risk of post-traumatic stress disorder, and worse quality of life (QoL) compared with general population norms. In a subset undergoing more extensive testing, some irregularities in neurocognitive functions were identified [15]. Rollins et al., evaluating patients with MIS-C at 6 to 12 months after discharge, found that they had worse working memory, more symptoms of depression, and a worse QoL than controls [16].

In line with these findings, the first cohort of 33 patients admitted to our hospital showed the presence of memory and attention difficulties, socio-emotional difficulties, and repercussions on QoL at 6 months of life [12].

The present study reports findings of a 12-month multidisciplinary follow-up in children with MIS-C, treated at a large tertiary-care paediatric hospital, with the aim of describing the trend of outcome measures from the acute phase through to 6 and finally 12 months of follow-up.

## Materials and methods

### Study design and population

A retrospective cohort study was conducted among all children and adolescents (aged ≤ 18 years) with a diagnosis of MIS-C meeting the CDC criteria who were hospitalised between October 1, 2020, and May 31, 2022, at the Buzzi Children’s Hospital, a large tertiary-care referral paediatric hospital in Milan.

All data were collected in a dedicated database and retrospectively analysed and processed in the period October 2022–June 2023.

Patients were managed and followed up by a multidisciplinary MIS-C team. All patients were treated during hospitalisation in accordance with our institutional multidisciplinary clinical protocol, already detailed elsewhere [12] and described in the supplementary material.

### Procedures

Multidisciplinary follow-up evaluations were scheduled at 6 and 12 months after admission. Patients also underwent cardiological evaluations at 1 week and at 1 month after admission, but the results of these are not discussed in this paper [7]. Details of the evaluations are provided in the supplementary material. Electronic clinical records were reviewed by two investigators (ARD and RDS), who collected baseline and follow-up data.

A panel of laboratory tests to explore systemic and organ-specific involvement was performed during the hospital stay and at each follow-up.

Cardiological, gastroenterological, and pneumological evaluations consisted of clinical and instrumental assessments (abdominal and lung imaging, echocardiograms). All echocardiography reports were reviewed by two senior expert cardiologists, specifically assigned to the care of MIS-C patients. Abnormal echocardiography results were defined as follows: abnormal ventricular function, e.g. ejection fraction < 0.55, coronary artery aneurysm or dilatation (z-score > 2), pericardial inflammation, or a combination of the above.

Metabolic and nutritional assessment consisted of anthropometric measurements, including weight and height, body mass index (BMI), arm and waist circumference, and triceps skinfolds. BMI (kg/m^2^) and the BMI z-score were calculated according to CDC growth chart reference values [17]. The homeostasis model analysis-insulin resistance (HOMA-IR) index, defined as (fasting plasma insulin (mU/L) × fasting plasma glucose (mg/dl)/405), was calculated. IR corresponded to a HOMA-IR value > 97th percentile for sex and pubertal stage [18].

The metabolic sequelae were classified as “never altered” if the HOMA index was normal at baseline and at the end of follow-up, “improved” if insulin resistance at baseline resolved during follow-up, and “maintained or worsened” if insulin resistance at baseline did not resolve during follow-up or insulin resistance occurred during follow-up.

The neurological assessment consisted of neurological examination and assessment of non-verbal IQ (Raven’s Progressive Matrices). In children who had previously been diagnosed with encephalitis, sleep and awake EEG were repeated at 6 and 12 months, while the Wechsler Intelligence Scale was administered at the 12-month follow-up.

**Fig. 1:**
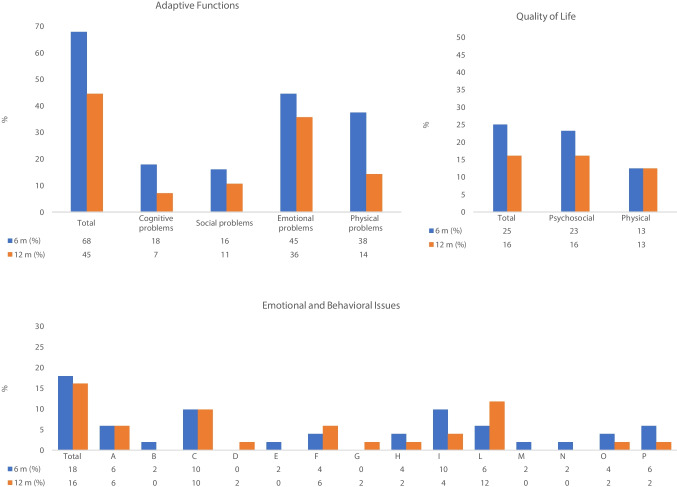
Well-being outcome measures at 6 and 12 months of follow-up. Adaptive functions: percentage of subjects reporting adaptive dysfunctions at 6 and 12 months of follow-up. *Cognitive problems* investigated: decreased performance/difficulty with tasks, attention deficit, memory impairment. *Social problems* investigated: school attendance, extracurricular activities, relational difficulties with peers, unwillingness to interact with peers or adults, unwillingness to play/spend time outside the home. *Emotional problems* investigated: irritability, tendency to complain, tantrums, regressive behaviour, self-harm, hyperactivity/attention deficit, low frustration tolerance, oppositional behaviour, excessive worrying over health, anxiety. *Physical problems* investigated: fatigue/tiredness, difficulty falling asleep, frequent awakenings/drowsiness during the day, tendency to complain of physical problems without medical causes. Quality of life: percentage of patients with pathological scores on the PedsQL questionnaire completed by parents at 6 and 12 months. Emotional and behavioural issues: percentage of patients with pathological scores on parent-reported CBCL questionnaire at 6 and 12 months after discharge. Columns: total, total pathological CBCL; subscales: A, anxious/depressed; B, emotionally reactive; C, withdrawn/depressed; D, somatic complaints; E, social problems; F, thought problems; G, attention problems; H, aggressive behaviour; I, affective problems; L, anxiety problems; M, pervasive development problems; N, somatic problems; O, ADHD problems; P, oppositional defiant problems

Questionnaires investigating adaptive functions, emotional and behavioural concerns, and QoL were used to explore general well-being 6 and 12 months after discharge:

- Adaptive functions, including cognitive (e.g. school performance), social (e.g. socialisation and return to school and leisure activities), psychological (e.g. anxiety), and physical problems (e.g. sleep disturbances and physical fatigue), were investigated through an ad hoc interview conducted with all patients and their parents (supplementary material); for the purpose of this research, we considered the presence/absence of symptoms related to any of the investigated domains.
- Emotional and behavioural issues were assessed using the parents’ version of the Child Behaviour Checklist (CBCL), and the results are reported in Fig. 1. Raw scores were converted to T-scores and considered pathological above the cut-off value of 70.
- QoL was assessed using the PedsQL (Pediatric Quality of Life Inventory™) interview validated for the Italian population and completed by parents. Both the PedsQL Total and the Generic Core Scales measuring psychological and physical health were considered [19]. Scores were considered clinically pathological for values below 75.

General well-being was defined as “good”, if no pathological scores were found on the various questionnaires, and “impaired”, if pathological scores were found on one or more of the three measures used (Adaptive Functions interview, CBCL, PedsQL). Trends in general well-being were classified as “never impaired” if the scores on the questionnaires were not pathological at baseline and at the end of follow-up, “improved” if the impaired general well-being at baseline resolved during follow-up, and “maintained or worsened” if the impaired general well-being at baseline did not resolve during follow-up, or if the impaired general well-being occurred during follow-up.

### Statistical analysis

The patients’ demographic and clinical characteristics and biochemical and instrumental data were collected in a dedicated database. Continuous variables are presented as mean values and 95% confidence intervals, and discrete variables as percentages. To test changes in the outcomes of interest over time, mixed-effects linear regression models were used. Time was included as a fixed-effect predictor and the patient as a random effect. The Bonferroni multiple comparison test was used to identify differences between time points. Generalised estimation equations with family binomial and link logit were used to estimate the prevalence of outcomes of interest over time. We used Fisher’s exact test to investigate the association between metabolic and neurological sequelae. Finally, we used Spearman’s correlation to assess associations between long-term sequelae and the acute phase clinical variables, e.g. ICU admission, length of hospital stay, age, laboratory biomarkers of inflammation (CRP, ferritin, D-dimer levels), and severity of neurological involvement (whether or not encephalitis had been diagnosed). A *p* value < 0.05 was considered statistically significant. Statistical analysis was performed using STATA version 12.0 (StataCorp).

## Results

Of the 62 patients admitted, 56 children and adolescents (40 M, 71.4%), with a mean age of 8.7 years (95% CI 7.7, 9.7), completed the follow-up at 12 months. Of the six who did not complete the follow-up, four could no longer be contacted after discharge, one moved abroad, and the other preferred not to attend follow-up appointments. Demographic data and clinical features of the cohort are shown in Fig. 1 Well-being outcome measures at 6 and 12 months of follow-up. Adaptive functions: percentage of subjects reporting adaptive dysfunctions at 6 and 12 months of follow-up. Cognitive problems investigated: decreased performance/difficulty with tasks, attention deficit, memory impairment. Social problems investigated: school attendance, extracurricular activities, relational difficulties with peers, unwillingness to interact with peers or adults, unwillingness to play/spend time outside the home. Emotional problems investigated: irritability, tendency to complain, tantrums, regressive behaviour, self-harm, hyperactivity/attention deficit, low frustration tolerance, oppositional behaviour, excessive worrying over health, anxiety. Physical problems investigated: fatigue/tiredness, difficulty falling asleep, frequent awakenings/drowsiness during the day, tendency to complain of physical problems without medical causes. Quality of life: percentage of patients with pathological scores on the PedsQL questionnaire completed by parents at 6 and 12 months. Emotional and behavioural issues: percentage of patients with pathological scores on parent-reported CBCL questionnaire at 6 and 12 months after discharge. Columns: total, total pathological CBCL; subscales: A, anxious/depressed; B, emotionally reactive; C, withdrawn/depressed; D, somatic complaints; E, social problems; F, thought problems; G, attention problems; H, aggressive behaviour; I, affective problems; L, anxiety problems; M, pervasive development problems; N, somatic problems; O, ADHD problems; P, oppositional defiant problems. Table 1 CRP and ferritin levels, frequently abnormal during the acute phase, had completely normalised at both follow-ups, while D-dimer levels, albeit showing a slow but progressive decrease, were still elevated in 12/56 (21.4%) at 6 months and in 2/56 (3.6%) at 12 months after admission. Mild anaemia was detected in 10/56 (17.9%) at 12 months after admission, while blood count alterations (neutrophilia, lymphopenia, and thrombocytopenia) were normal at 6 months and 12 months of follow-up (Table 2).

**Table 1:** Demographic data and clinical characteristic at hospitalisation

Patients, *N* (M%)	40/56 (71.4%)
Age at diagnosis (range)	8.7 (2–17 years)
Ethnicity	
Caucasian (%)	41 (73.2%)
Black (%)	3 (5.4%)
Asian (%)	7 (12.5%)
Hispanic (%)	5 (89%)
BMI	
Underweight (%)	4 (7.1%)
Normal range (%)	38 (67.9%)
Overweight (%)	11 (19.6%)
Obese (%)	3 (5.4%)
SARS-CoV-2 PCR-positive at admission (%)	6/56 (10.7%)
SARS-CoV2 IgG-positive at admission (%)	49/49 (100%)
Vaccinated against SARS-CoV-2 at admission (%)	5 (8.9%)
Length of hospital stay, days (IQR)	13.2 (10.75–16)
Non-invasive ventilation (High Flow Nasal Cannula or nasal CPAP)	12 (21.4%)
Paediatric ICU admission	34 (60.7%)
Therapy	
Intravenous immunoglobulin 2 g/kg	56 (100%)
Intravenous methylprednisolone	54 (96.4%)
High dose	11 (19.6%)
Intermediate dose	13 (23.3%)
Low dose	30 (53.6%)
Aspirin	56 (100%)
Enoxaparin	50 (89.3%)
Inotropic support	17 (30.3%)

**Table 2:** Organ involvement at admission (TO) and after 6 (T1) and 12 months (T2)

	**T0**	**T1**	**T2**
Blood cell count abnormalities			
Anaemia	49/56 (87.5%)	4/56 (7.1%)	10/56 (17.8%)
Neutrophilia	27/56 (48.2%)	1/56 (1.8%)	2/56 (3.6%)
Lymphopenia	51/56 (91.1%)	0/56 (0%)	0/56 (0%)
Thrombocytopenia	21/56 (37.5%)	2/56 (3.6%)	1/56 (1.8%)
Systemic inflammatory markers			
Elevated C-reactive protein	56/56 (100%)	0/56 (0%)	0/56 (0%)
Elevated ferritin	56/56 (100%)	0/56 (0%)	0/56 (0%)
Elevated D-dimer	56/56 (100%)	12/56 (21.4%)	2/56 (3.6%)
Heart involvement			
Reduced LVEF	35/56 (62.5%)	0/56 (0%)	0/56 (0%)
Mildly reduced LVEF	20/56 (35.7%)	0/56 (0%)	0/56 (0%)
Moderately LVEF	9/56 (16.1%)	0/56 (0%)	0/56 (0%)
Severely LVEF	6/56 (10.7%)	0/56 (0%)	0/56 (0%)
Elevated troponin T	26/56 (46.4%)	0/56 (0%)	0/56 (0%)
Elevated NT-proBNP	51/55 (91.1%)	0/56 (0%)	0/56 (0%)
Lung involvement			
Respiratory symptoms	20/56 (35.7%)	16/56 (28.6%)	9/56 (16.1%)
Imaging abnormalities	22/56 (39.3%)	0/56 (0%)	0/56 (0%)
Gastrointestinal involvement			
Abdominal symptoms	50/56 (89.3%)	0/56 (0%)	0/56 (0%)
Imaging abnormalities	38/56 (67.9%)	0/56 (0%)	0/56 (0%)
Renal involvement and electrolytes			
Elevated creatinine	16/56 (28·6%)	0/56 (0%)	0/56 (0%)
Hypoalbuminemia	56/56 (100%)	0/56 (0%)	0/56 (0%)
Hyponatremia	38/56 (68%)	0/56 (0%)	0/56 (0%)
Hypokalemia	24/56 (43%)	0/56 (0%)	0/56 (0%)
Metabolic markers			
Hypertrigliceridemia	46/52 (88%)	8/56 (14%)	3/56 (5%)
Impaired fasting glucose	33/55 (60%)	1/56 (2%)	2/56 (4%)
Neurological involvement			
Neurological signs	41/56 (73%)	0/56 (0%)	0/56 (0%)
Encephalopathy	17/56 (30%)	0/56 (0%)	0/56 (0%)
EEG abnormalities	19/56 (34%)	0/56 (0%)	0/56 (0%)

Anaemia (< 2 years: Hb < 10.5 g/dl; 2–12 years: < 11.5; 12–18 years: < 12 -girls-, < 13 -boys-); neutrophilia (neutrophils ≥ 7000/mmc); lymphopenia (lymphocytes ≤ 2000/mmc); thrombocytopenia (thrombocytes ≤ 150,000/mmc); elevated C-reactive protein (≥ 10 mg/l); elevated ferritin (≥ 140 µg/l); elevated D-dimer (≥ 500 µg/l); reduced LVEF (FE < 55%); mildly reduced LVEF (FE 45–54%); moderately reduced LVEF (FE 36–44%); severely reduced LVEF (FE ≤ 35%); elevated troponin T (≥ 30 ng/l); elevated NT-proBNP (≥ 450 ng/l); elevated creatinine (< 1 year: > 0.4 mg/dl; 1–3 years: > 0.35; 3–7 years: > 0.45; 7–11 years: > 0.6; 11–18 years: > 0.75); hypoalbuminemia (albumin ≤ 3 g/dl); hyponatremia (Na +  < 135 mEq/l); hypokalemia (K +  < 3.5 mEq/l); hypertrigliceridemia (≥ 130 mg/dl 10–19 years, ≥ 100, 0–9 years); impaired fasting glucose (100–125 mg/dl).· LVEF, left ventricular ejection fraction

At both 6 and 12 months, all signs of cardiac and gastrointestinal involvement had completely disappeared in all the patients. No changes on lung ultrasound imaging were detected at follow-up in any patient, while respiratory symptoms such as dyspnoea on exertion and occasional asthenia were found in 16/56 (28.6%) at 6 months and in 9/56 (16.1%) at the 12-month follow-up (Table 2).

At admission, the prevalence rates of overweight and obesity were 19.6% and 5.4%, respectively, whereas 7.1% of the patients were underweight. The mean BMI z-score was 0.08 (53rd perc.) at admission, and 0.6 (72nd perc.) both at 6 and at 12 months. The BMI z-score was significantly increased at 6 months versus admission (+ 0.526, 95% CI 0.284, 0.767, *p* < 0.001) (Table 3), and this was maintained at 12 months of follow-up. Abnormal HOMA-IR index and TyG index during hospitalisation were observed in 92.1% and 98.1% of the sample, respectively (Table 4). At 6 months, HOMA-IR index and TyG index values were found to be abnormal in 31.1% and in 71.4% of the population, respectively. TyG index showed a further significant reduction at the 12-month versus the 6-month follow-up (− 0.34, 95% CI − 0.53, − 0.19, *p* < 0.001), but both these indices were still pathological in about a third of the population at 12 months. Overall, 5.6% of subjects never presented insulin resistance, and 61.1% despite having abnormal HOMA values at baseline resolved at the end of follow-up. The remaining 33.3%, on the contrary, did not resolve or worsen insulin resistance. All the other metabolic parameters (glucose, insulin, triglycerides) decreased significantly over time, already showing complete normalisation at 6 months (Table 3).

**Table 3:** Anthropometric, glycometabolic, and inflammatory parameters at hospitalisation (T0) and after 6 (T1) and 12 months (T2) of follow-up

Variable	T0 Mean (95% CI)	T1 Mean (95% CI)	T2 Mean (95% CI)
BMI z-score	0.078 (− 0.217, 0.372)_a_	0.603 (0.308, 0.898)_b_	0.598 (0.304, 0.893)_b_
Glucose (mg/dl)	111 (106, 115)_a_	86 (82, 92)_b_	86 (82, 91)_b_
Insulin (U/l)	20.2 (17.0, 23.4)_a_	12.1 (9.4, 14.8)_b_	11.4 (8.7, 14.1)_b_
Triglycerides (mg/dl)	203 (184, 223)_a_	79 (60, 97)_b_	65 (46, 83)_b_
D-dimer (µg/l)	6233 (4863, 7602)_a_	527 (− 843, 1897)_b_	309 (− 1061, 1679)_b_
CRP (mg/l)	192.1 (177.2, 207)_a_	1.5 (− 13.4, 16.4)_b_	1.3 (− 13.6, 16.2)_b_

Values are marginal means and 95% confidence intervals obtained from a mixed-effects linear model. Different subscript letters indicate significant difference at Bonferroni multi-comparison post-hoc test

**Table 4:** Mean values of glycometabolic parameters and prevalence of abnormal HOMA-IR index and TyG index at hospitalisation (T0), after 6 (T1) and 12 months (T2) of follow-up

	T0	T1	T2	*p* value	*p* value T1 vs. T0	*p* value T2 vs. T0	*p* value T2 vs. T1
HOMA-IR index mean* (95% CI)	5.74 (4.83, 6.66)a	2.63 (1.85, 3.40)b	2.50 (1.72, 3.28)b	< 0.001	< 0.001	< 0.001	1.000
TyG index mean* (95% CI)	9.15 (9.01, 9.28)a	8.04 (7.92, 8.17)b	7.80 (7.67, 7.93)c	< 0.001	< 0.001	< 0.001	0.019
Abnormal HOMA-IR index %^ŧ^ (95% CI)	92.0 (83.3, 1.00)	31.1 (18.6, 43.5)	32.1 (19.4, 44.8)	< 0.001	< 0.001	< 0.001	0.889
Abnormal TyG index %^ŧ^ (95% CI)	98.1 (94.4, 1.00)	71.4 (59.5, 83.4)	35.7 (23.1, 48.4)	< 0.001	0.006	< 0.001	< 0.001

^*^Values are marginal means and 95% confidence intervals (in brackets) obtained from a mixed-effects linear model.· Different subscript letters indicate significant difference at Bonferroni multi-comparison post-hoc test

^ŧ^Values are marginal probabilities and 95% confidence intervals (in brackets) obtained from generalised estimation equation with family binomial and link logit· abnormal HOMA-IR index (> 97th perc· for sex and age); abnormal TyG index (≥ 7·88)

Neurological assessment and Raven’s Progressive Matrices gave normal results in all the subjects, as did the EEG and Wechsler Intelligence Scales, performed in the children who had had encephalitis. Impairment of adaptive functions after discharge was reported, by parents, in 67.9% of patients at 6 months and in 44.6% at 12 months.

Pathological total scores on the CBCL were found in 17.9% of patients at 6 months and in 16.1% at 12 months, while the PedsQL questionnaire returned pathological scores in 25% of patients at 6 months and in 16.1% at 12 months. The PedsQL psycho-social health score was pathological in 23.2% of patients at 6 months and in 16.1% at 12 months, while the physical health score was pathological in 12.5% of patients at 6 months and in 12.5% at 12 months (Fig. 1). Overall, 25.0% of subjects always presented with general well-being, and 28.6%, although they had pathological scores in one or more questionnaires at 6 months, resolved in the next 6 months of follow-up. The remaining 46.4%, on the contrary, did not resolve or report pathological scores at 12 months.

Analysis of the trends shown by the above measures revealed that significantly fewer patients presented adaptation difficulties at 12 months compared with 6 months (*p*, 0.003). Conversely, no significant difference was observed in the number of children with emotional and behavioural issues or a reduced QoL at 12 versus 6 months of follow-up (Table 5).

**Table 5:** Changes in the well-being outcomes at 12 months (T2) versus 6 months (T1) of follow-up

	T1% (95% CI)	T2% (95% CI)	*p* value
Adaptive functions	67.9 (55.5, 80.2)	44.6 (31.5, 57.8)	0.003
Quality of life	25.0 (13.6, 36.4)	17.9 (7.7, 28.0)	0.157
Emotional and behavioural issues	17.9 (7.7, 28.0)	16.1 (6.4, 25.8)	0.657

Values are marginal probabilities and 95% confidence interval (in bracket) obtained from logistic regression

With the exception of the HOMA-IR index at admission, no significant correlations emerged between the acute phase variables analysed (ICU admission, length of hospital stay, age, laboratory markers of inflammation—CRP, ferritin, D-dimer levels—severity of neurological impairment) and the presence of long-term sequelae.

Finally, the following association was found between metabolic sequelae and the measures of general well-being: in the patients whose IR either persisted or worsened during follow-up, compared with those whose HOMA-IR index values normalised, we documented a higher prevalence of subjects whose general well-being remained stable or worsened between 6 and 12 months of follow-up (75% vs. 40.9% *p* < 0.001).

## Discussion

Fifty-six children with MIS-C, hospitalised between October 1, 2020, and May 31, 2022, at the Buzzi Children’s Hospital in Milan, were followed up by a multidisciplinary team for 12 months following their admission to hospital. Our tertiary-care paediatric hospital was the referral hub for MIS-C in Lombardy, which was among the European regions hardest hit by the SARS-CoV-2 pandemic.

The multidisciplinary follow-up of these patients revealed no long-term effects on the heart and lungs, in agreement with the available literature [10, 20]; it also showed complete resolution of the neurological symptoms that characterised the clinical picture during hospitalisation [21]. By 6 months post-admission, there was already almost complete resolution of the clinical, laboratory, and instrumental abnormalities observed in the acute inflammatory phase, and by the end of the follow-up, these abnormalities had completely disappeared.

In contrast, we observed increased BMI values (e.g. increased adipose tissue) 6 months after the acute phase [22]. This finding is in agreement with literature reports [9, 11, 13].

Furthermore, at 12 months, a significant portion of children showed weight gain compared with their BMI at admission, as confirmed by increased BMI z-scores (Table 3). The finding of BMI z-score increases at 6 and 12 months after admission may be due to a lack of physical activity and/or to the presence of anxiety and/or depressive symptoms/emotional problems, factors that may reinforce each other and play a role in increasing BMI.

HOMA-IR index and TyG index were pathological in most of children during the acute phase. The release of a wide spectrum of cytokines, including IL-6 and tumour necrosis factor (TNF)-α, during the acute phase of MIS-C, leads to a systemic proinflammatory milieu [23]. Increased levels of IL-6 and TNF-α, in particular, are known to contribute to IR and b-cell hyperstimulation [24]. Despite of almost complete inflammatory biomarkers normalisation at mid- and long-term follow-up, HOMA-IR index and TyG index were found still abnormal in 31.1% and 71.4% of patients, respectively, at 6 months. Notably, the condition of IR persisted in about a third of the population at 12 months.

Of all the factors influencing the adverse effects of systemic glucocorticoids, dose and duration of therapy are the most important independent and well-documented risk factors [25]. Overall, it can be stated that prolonged exposure is a high-risk factor [26]. In regard to the long-term metabolic effects observed in our study, although the effects of systemic corticosteroids cannot be totally ruled out, it should be noted that only 20% of our patients were treated with high dose of systemic steroids, while we observed the persistence of IR in about one-third of patients. Moreover, metabolic adverse effects due to systemic corticosteroids are mostly associated with the long-term duration treatment (3 months or more) [27, 28]. Last, but not least, our patients were treated with systemic corticosteroids with intermediate biologic half-lives (e.g. methylpredinosolone and prednisone) that have lower potential for side effects than analogues do with long biologic half-lives (e.g. betamethasone) [29]. All these reasons make it unlikely that treatment with systemic corticosteroids may have influenced the long-term metabolic outcome in our study.

On the other hand, endocrine and metabolic system damage, directly or indirectly attributable to viral infection, seems to persist over time and to contribute to the long-term IR, as shown by preliminary data in our cohort [30].

The other sequelae observed in our patients can be grouped as signs and symptoms liable to affect general well-being. Problems of this kind were present at 6 months post-admission in the majority of patients and consisted mostly of a decline in adaptive functions, as shown by regressive behaviours, difficulty coping with usual school and play activities, fatigue, sleep problems, irritability, and excessive worrying about their health. About a fifth of the children also showed clinical repercussions, characterised by behavioural and emotional issues, and low QoL. This same clinical profile has already been observed and described in the first group of children treated at our hospital [12] and is confirmed by the present follow-up in the entire study sample. While the adaptive problems were resolved by 12 months in most of the children, the behavioural and emotional difficulties and low QoL, although rarer, persisted over time, with similar rates found between the 6- and 12-month assessments. Finally, it is noted that none of the children who were asymptomatic at 6 months developed symptoms during the remainder of the follow-up.

Our observations confirm the findings of other studies documenting the presence of emotional and behavioural problems in association with a lower QoL in children who presented MIS-C [13, 15]. It is possible that the experience of being ill and hospitalised might, in itself, explain, completely or in part, the appearance of this set of symptoms. Although this is still a new concept, recent evidence suggests that a severe and unexpected illness may act as a health risk factor when experienced by otherwise healthy individuals (of which children with MIS-C are but one example). In fact, a scenario of this type has recently been described in children hospitalised in intensive care. The “post-intensive care unit syndrome in paediatrics” developed by these subjects is defined as a constellation of cognitive, physical, and mental health impairments occurring after ICU admission, which may reflect changes in brain function (e.g. new-onset neurological morbidity) or in family dynamics, or physical changes [15, 31]. To further investigate this emerging concept, a structured follow-up protocol for children in the ICU has, in some countries, been introduced into national guidelines, the aim being to evaluate interventions designed to mitigate the risk of poor outcomes and therefore optimise the health of the child. In our sample, these impairments were observed both in children admitted to the ICU and in ones treated on regular wards; this, together with the fact that no correlation emerged with the length of ICU stay, seems to suggest that patients do not have to be admitted to the ICU in order to experience long-term effects of illness/hospitalisation on their psychological health and quality of life.

That said, although metabolic and psychosocial problems are relatively frequent 6 months after the physical and emotional destabilisation associated with the acute event, the majority of children had a positive long-term outcome, with no significant impact on their physical, mental, and social well-being. Nevertheless, at 1 year, a not negligible proportion still presented metabolic and emotional-behavioural alterations and a reduction in their QoL. Moreover, an overlap was found between the subset of patients with metabolic issues and those with problems liable to affect their general well-being and QoL.

To our best knowledge, this is the first study reporting long-term glycometabolic alterations, joined to persistent impairment in the realm of general well-being, behaviour, and decline in QoL, in a subgroup of children previously hospitalised with MIS-C.

Neither the severity of the inflammatory markers during the acute phase nor the duration of hospitalisation seems to be correlated either with the medium-term or with the long-term outcome. However, it remains conceivable that the acute illness per se may act as a trigger factor in a subgroup of predisposed subjects. MIS-C itself appears to develop in genetically predisposed individuals [32–34].

The presence of IR has been associated with neurocognitive impairments affecting, for example, executive functions and memory [35]. The endothelial dysfunctions caused by IR may result in blood–brain barrier breakdown, which in turn may allow proinflammatory molecules from the plasma to penetrate the CNS, thereby contributing to neuronal abnormalities and neuroinflammation [36]. Finally, the long-term sequelae of MIS-C may, in some subjects, also be due to the persistence SARS-CoV-2 in adipose tissue or other sites [37].

However, the mechanisms that might explain the long-term persistence of both metabolic alterations and neuro-behavioural outcomes and their possible relationship are far from being clarified.

Nevertheless, these findings highlight the possible impact of MIS-C on long-term health, understood not merely (or not so much) as the absence of disease or infirmity, but rather as physical, mental, and social well-being, in accordance with the WHO definition.

Our study has particular strengths, such as the presence of an MIS-C-dedicated multidisciplinary team which carried out serial and standardised clinical and instrumental assessments, and the fact that, since our centre served as a hub during the pandemic, we were able to collect a good-sized sample.

Weaknesses were the lack of a control group and the absence of a reliable pre-MIS-C well-being profile with which to compare the follow-up data relating to the psychological and emotional health, adaptive functions, and QoL of the enrolled children.

These findings point out to the potential long-term effects of pandemics and to the importance of a multidisciplinary follow-up to detect potential negative sequelae in different areas of health, both physical and psychosocial.

In particular, there is a need to have studies that examine the long-term effects of this complication on patients that have had MIS-C.

## Supplementary Information

Below is the link to the electronic supplementary material.

Supplementary file1 (DOCX 33 KB)

## Abbreviations

BMI: Body mass index
CBCL: Child Behaviour Checklist
CDC: Centers for Disease Control and Prevention
CNS: Central nervous system
CRP: C-reactive protein
EEG: Electroencephalogram
ENT: Ear, nose and throat
HOMA-IR: Homeostasis model analysis-insulin resistance
ICU: Intensive care unit
IL-6: Interleukin-6
IQ: Intelligence quotient
MIS-C: Multisystem inflammatory syndrome in children
PCR: Polymerase chain reaction
PedsQL: Pediatric Quality of Life Inventory™
QoL: Quality of life
TNF: Tumour necrosis factor
TyG: Triglycerides
WHO: World Health Organisation
