# Supplementary material for: Long−term health outcome and quality of life in children with multisystem inflammatory syndrome: findings from multidisciplinary follow−up at an Italian tertiary−care paediatric hospital
Source: Eur J Pediatr. 2024 Sep 10;183(11):4885–95. doi: 10.1007/s00431-024-05706-0 (PMC11473591; doi:10.1007/s00431-024-05706-0)
Supplement: Supplementary file 1 — Supplementary file1 (DOCX 33 KB) [file 431_2024_5706_MOESM1_ESM.docx]

**Title page**

**^Long-term health outcome and quality of life in children with multisystem inflammatory syndrome: findings from multidisciplinary follow up at an Italian tertiary-care paediatric hospital^**

Enza D’Auria^1*^, Stefania Maria Bova^2^*, Andrea Riccardo Dallapiccola^3^, Raffaella De Santis^3^, Alessandro Leone^4+5^, Valeria Calcaterra^1+6^, Savina Mannarino^7^, Massimo Garbin^7^, Sara Olivotto^2^, Salvatore Zirpoli^8^, Michele Ghezzi^1^, Alice Marianna Munari^8^, Elvira Verduci^1+3^, Andrea Farolfi^1^, Alessandra Bosetti^1^, Veronica Perico^1^, Pietro Capetti^3^, Arianna Gadda^3^, Laura Gianolio^3^, Germana Lo Monaco^3^, Luisa Lonoce^3^, Roberto Previtali^2+3^, Ludovica Serafini^3^, Silvia Taranto^3^, Pierangelo Veggiotti^2+3^, Gianvincenzo Zuccotti^1+3^

*^1^ Department of Paediatrics, Buzzi Children’s Hospital, ASST-FBF-Sacco, Milan, Italy*

*^2^ Paediatric Neurology Unit, Buzzi Children’s Hospital, Milan, Italy*

*^3^ Department of Biomedical and Clinical Science, University of Milan, Milan, Italy*

*^4^ International Center for the Assessment of Nutritional Status and the Development of Dietary Intervention Strategies (ICANS-DIS), Department of Food, Environmental and Nutritional Sciences (DeFENS), University of Milan, 20133 Milan, Italy.*

*^6^ IRCCS Istituto Auxologico Italiano, Clinical Nutrition Unit, Department of Endocrine and Metabolic Medicine, Milan, Italy*

*^6^ Paediatric and Adolescent Unit, Department of Internal Medicine and Therapeutics, University of Pavia, Pavia, Italy*

*^7^ Paediatric Cardiology Unit, V. Buzzi Children’s Hospital, Milan, Italy*

*^8^ Department of Pediatric Radiology and Neuroradiology, Buzzi Children’s Hospital, Milan, Italy*

** ED and SMB contributed equally.*

Enza D’Auria MD, PhD: [enza.dauria@unimi.it](mailto:enza.dauria@unimi.it); 0000-0003-2750-5810
Stefania Maria Bova MD: [stefania.bova@asst-fbf-sacco.it](mailto:stefania.bova@asst-fbf-sacco.it); 0000-0002-4092-7412
Andrea Riccardo Dallapiccola MD: [andrea.dallapiccola@unimi.it](mailto:andrea.dallapiccola@unimi.it); 0000-0002-7471-5733
Raffaella De Santis MD: [raffaella.desantis@unimi.it](mailto:raffaella.desantis@unimi.it); 0009-0002-8488-1185
Alessandro Leone PhD: [alessandro.leone1@unimi.it](mailto:alessandro.leone1@unimi.it); 0000-0001-8063-8490
Valeria Calcaterra MD, PhD: [valeria.calcaterra@asst-fbf-sacco.it](mailto:valeria.calcaterra@asst-fbf-sacco.it); 0000-0002-2137-5974
Savina Mannarino MD: [savina.mannarino@asst-fbf-sacco.it](mailto:savina.mannarino@asst-fbf-sacco.it); 0000-0001-9099-1845
Massimo Garbin MD: [massimo.garbin@asst-fbf-sacco.it](mailto:massimo.garbin@asst-fbf-sacco.it); 0000-0002-1694-2559
Sara Olivotto MD: [sara.olivotto@asst-fbf-sacco.it](mailto:sara.olivotto@asst-fbf-sacco.it); 0000-0003-2612-1727
Salvatore Zirpoli MD: [salvatore.zirpoli@asst-fbf-sacco.it](mailto:salvatore.zirpoli@asst-fbf-sacco.it); 0000-0002-1790-4618
Michele Ghezzi MD: [michele.ghezzi@asst-fbf-sacco.it](mailto:michele.ghezzi@asst-fbf-sacco.it); 0000-0002-7434-9112
Alice Marianna Munari MD: [alice.munari@asst-fbf-sacco.it](mailto:alice.munari@asst-fbf-sacco.it); 0000-0001-5117-6530
Elvira Verduci^1^Prof., MD: [elvira.verduci@unimi.it](mailto:elvira.verduci@unimi.it); 0000-0003-2111-3111
Andrea Farolfi MD: [andrea.farolfi@asst-fbf-sacco.it](mailto:andrea.farolfi@asst-fbf-sacco.it); 0000-0002-0953-2551
Alessandra Bosetti MSc: [alessandra.bosetti@asst-fbf-sacco.it](mailto:alessandra.bosetti@asst-fbf-sacco.it); 0009-0006-8062-3268
Veronica Perico MSc: [veronica.perico@unimi.it](mailto:veronica.perico@unimi.it); 0000-0001-7741-0874
Pietro Capetti MD: [pietro.capetti@unimi.it](mailto:pietro.capetti@unimi.it); 0000-0003-0647-4170
Arianna Gadda MD: [arianna.gadda@unimi.it](mailto:arianna.gadda@unimi.it); 0000-0001-6184-3413
Laura Gianolio MD: [laura.gianolio@unimi.it](mailto:laura.gianolio@unimi.it); 0009-0003-1189-9373
Germana Lo Monaco MD: [germana.lomonaco@unimi.it](mailto:germana.lomonaco@unimi.it); 0009-0000-8595-6092
Luisa Lonoce MD: [luisa.lonoce@unimi.it](mailto:luisa.lonoce@unimi.it); 0000-0002-8768-2993
Roberto Previtali MD: [roberto.previtali@unimi.it](mailto:roberto.previtali@unimi.it); 0000-0002-3295-0655
Serafini Ludovica MD: [ludovica.serafini@unimi.it](mailto:ludovica.serafini@unimi.it); 0000-0003-2750-5810
Taranto Silvia MD: [silvia.taranto@unimi.it](mailto:silvia.taranto@unimi.it); 0009-0007-8113-5829
Pierangelo Veggiotti Full Professor: [pierangelo.veggiotti@unimi.it](mailto:pierangelo.veggiotti@unimi.it); 0000-0003-2851-3441
Gianvincenzo Zuccotti Full Professor: [gianvincenzo.zuccotti@unimi.it](mailto:gianvincenzo.zuccotti@unimi.it); 0000-0002-2795-9874

Correspondence to:

Dr. Enza D’Auria, Department of Pediatrics, Buzzi Children’s Hospital, ASST-FBF-Sacco,

Via Castelvetro 32, Milan, Italy.

enza.dauria@asst-fbf-sacco.it

**Multidisciplinary assessment and therapy**

1. **Acute phase:**

- **Clinical evaluation:** a complete anamnesis and a paediatric physical examination were performed to identify multisystem organ involvement. The physical examination included evaluation of pubertal stage and anthropometric measurements of *weight and height* (measured using a mechanical column scale with an altimeter Seca 711), *arm and waist circumferences* (measured with a Seca 201 tape measure), and *tricipital skinfolds* (measured using a Holtain 610 caliper). Also, *BMI* (kg/m2), and the *BMI Z-Score* were established according to CDC growth chart reference values ^[1]^.
- **Laboratory assessment,** including:
  - *Inflammatory marker*s: ESR, CPR, procalcitonin, ferritin, LDH, IL-6, blood cell count, fibrinogen, D-dimer, PT and aPTT
  - *Metabolic panel*: liver, renal and pancreatic function, electrolytes, total and HDL cholesterol, fasting plasma glucose (FPG), and triglycerides (TG)
  - *Cardiac markers*: CPK, troponin, proBNP
  - *Endocrinological profile* performed within 24h of admission: free T3 (FT3), free T4 (FT4) and TSH, fasting plasma insulin (FPI), cortisol, and ACTH levels.
  - *SARS-CoV-2 RT-PCR test and serology*
  - *Urine test*
  - Other cultures, serology and viral PCR were performed to exclude differential diagnosis.

- **Cardiologic assessment**:
  - *Echocardiography:* left ventricular (LV) function was qualitatively and quantitatively assessed using a Philips Affinity 70 or Vivid S5 GE Healthcare, and the left ventricular ejection fraction (LVEF) was calculated according to Simpson’s biplane method. Left cardiac function was classified as follows: normal (LVEF > 55%), mildly–moderately reduced (LVEF ≤45%), or severely reduced (LVEF ≤35%). Mitral regurgitation (qualitative and semi-quantitative evaluation), the degree of pericardial effusion (mild if ≤5 mm, moderate if >5 mm), and coronary artery size (Montreal Z-score) were also measured [2].
  - *Electrocardiogram:* to exclude cardiac electric abnormalities and arrhythmias
- **Pneumological assessment**:
  - *Chest x-ray*, generally within 24 hours of admission. Pulmonary involvement was defined by the presence of pulmonary opacities, peri-bronchial thickening, pleural effusion and/or cardiomegaly; a simplified version of the Radiographic Assessment of Lung Edema Score was used, assigning a score of 0–4 to each lung depending on the extent of involvement (0 = no involvement; 1 = <25%; 2 = 25%–50%; 3 = 50%–75%; 4 = >75% involvement) [3].
  - *Lung ultrasonography* (US), after IVIG therapy: each lung was divided into 6 regions, and 4 patterns of involvement were defined according to global lung US aeration score: normal (lung sliding and A-lines - 0 points); multiple well-defined vertical B-lines extending from the pleural line or a small subpleural consolidation (1 point); presence of multiple confluent vertical B-lines extending from the pleural line or a small subpleural consolidation (2 points); presence of lung consolidation with air bronchograms (3 points) [4].
- **Gastroenterological evaluation**:
  - *Abdominal ultrasound*: ascites, mesenteric lymphadenitis, abnormalities of the appendix and gallbladder (increased wall thickening and/or stones) and increased ileal and/ or cecal wall thickening were considered pathological.
- **Neurological assessment**:
  - *Neurological examination*: carried out by a child neurologist
  - *Electroencephalogram (EEG)*: performed with wake and sleep recording in subjects with altered mental status
  - *Brain magnetic resonance imaging* (*MRI*): in subjects with encephalopathy and EEG abnormalities
  - *Cerebrospinal fluid (CSF) sampling*, including SARS-CoV-2 PCR and neurotropic viral PCR, isoelectrofocusing and CSF IgG, IgM and light chains (in subjects with EEG abnormalities)

**Therapy protocol**

- The cardinal treatment consisted of IVIG at 2 g/kg. In the event of haemodynamic impairment, need for oxygen, and heart failure, corticosteroids (methylprednisolone) were added. In the event of mildly reduced LVEF or minimal oxygen support, we gave methylprednisolone 2 mg/kg for 5 days, followed by gradual tapering. If there was a significant oxygen requirement, mild organ injury, and/or moderately reduced LVEF, methylprednisolone 10 mg/kg for one day then 2 mg/kg for 5 days was given before tapering. If there was a need for respiratory support, inotropic support, or if there was moderate-to-severe organ damage, and/or severe heart dysfunction, and/or neurological involvement, we used a high methylprednisolone dose of up to 30 mg/kg for 3 days.
- Supportive care (inotropes, fluid resuscitation, diuretics, oxygen and ventilation, antibiotics, anticoagulation, or anti-thrombotic prophylaxis) was considered in all the patients, according to clinical evaluation by the multidisciplinary team. The anti-thrombotic prophylaxis was started in all patients >12 years old, and was considered in patients <12 years old, if the D-dimer was high (>5 times the upper normal value) or if there was at least one known risk factor for thromboembolism. The anticoagulation therapy was prescribed in the presence of thrombosis and in the event of severe LV dysfunction. With reduction of the D-dimer or at normalization of LV function, heparin was replaced with low-dose aspirin for 3–4 weeks.

1. **Follow up at six and 12 months*:***

- **Clinical evaluation:** a complete anamnesis and ad hoc structured interview were performed, focusing on persistence of symptoms after hospitalisation (dyspnoea, asthenia, recurrent respiratory infections and/or abdominal pain), resumption of physical activity, change of eating habits and relationship with food. A complete paediatric physical examination also included evaluation of pubertal stage, anthropometric measurements and BMI calculation.
- **Laboratory assessment,** including:
  - *Inflammatory marker*s: ESR, CPR, ferritin, LDH, blood cell count, fibrinogen, D-dimer, PT and aPTT
  - *Metabolic panel*: liver, renal and pancreatic function, electrolytes, total and HDL cholesterol, fasting plasma glucose (FPG), and triglycerides (TG)
  - *Cardiac markers*: CPK, troponin
  - *Endocrinological profile*: free T3 (FT3), free T4 (FT4) and TSH, fasting plasma insulin (FPI), cortisol, and ACTH levels.
  - *SARS-CoV-2 serology*
- **Cardiological assessment**:
  - *Echocardiography*: to evaluate left ventricular (LV) function and its degree (normal: LVEF > 55%, mildly–moderately reduced: LVEF ≤45%, or severely reduced: LVEF ≤35%); mitral regurgitation (qualitative and semi-quantitative evaluation), pericardial effusion (mild if ≤5 mm, moderate if >5 mm), and coronary artery size (Montreal Z-score)
  - *Electrocardiogram*: to exclude cardiac electric abnormalities and arrhythmias
- **Pneumological assessment**:
  - *Lung ultrasonography* (US): see above
  - *Pulmonary function tests*: Forced vital capacity (FVC), forced expiratory volume in 1 second (FEV1), and forced expiratory flow during the middle half of FVC (FEF_25–75_) were measured by spirometry and the FEV1/FVC ratio was calculated.
- **Gastroenterological evaluation**:
  - *Abdominal ultrasound* (US): see above
- **Neurological assessment**:
  - *Neurological examination*
  - *Cognitive functions*:
    - Raven’s Progressive Matrices
    - Wechsler Intelligence Scale at 12 months in subjects with previous encephalitis
  - *EEG* - asleep and awake - in subjects with previous encephalitis.
- **Adaptive functions:** *ad hoc* interview conducted with all patients and their parents investigating the following domains in the last 6 months (box):
  - *Cognitive problems* investigated: decreased performance/difficulty with tasks, attention deficit, memory impairment.
  - *Social problems* investigated: school attendance, extracurricular activities, relational difficulties with peers, unwillingness to interact with peers or adults, unwillingness to play/spend time outside the home.
  - *Emotional problems* investigated: irritability, tendency to complain, tantrums, regressive behaviour, self-harm, hyperactivity/attention deficit, low frustration tolerance, oppositional behaviour, excessive worrying over health, anxiety.
  - *Physical problems* investigated: fatigue/tiredness, difficulty falling asleep, frequent awakenings/drowsiness during the day, tendency to complain of physical problems without apparent medical causes.
- **Emotional and behavioural concerns:** Child Behavior Checklist, parents’ questionnaire (CBCL);
- **Quality of Life**: The PedsQL—Pediatric Quality of Life Inventory™

| **ADAPTIVE FUNCTIONS INTERVIEW** |
| --- |
| **Have any of these conditions ever been present in the last 6 months?** |
| - Decline in academic performance, difficulty doing homework or following lessons (reported by teachers or otherwise)? |
| - Increased distractibility and poor attention to activities? |
| - Memory disorder? |
| - Difficulty in regularly returning to school? |
| - Difficulty in regularly resuming extracurricular activities? |
| - Relationship difficulties with peers and at school (bullying and the like)? |
| - Little desire to interact with peers or adults? |
| - Little desire to play or spend time outside the home? |
| - Irritability, tendency to complain, tantrums? |
| - Regressive behaviours (co-sleeping, language regression, pacifier)? |
| - Signs of self-harm? |
| - Hyperactivity, greater need to move and talk? |
| - Fatigue, increased tiredness? |
| - Concern regarding health issues? |
| - Anxiety? |
| - Difficulty falling asleep, frequent awakenings, daytime drowsiness? |
| - Tendency to complain of physical ailments without apparent medical causes (e.g., stomach ache, headache, dizziness, pain in the limbs, general malaise...)? |

**References**

1. Growth Charts - Clinical Growth Charts. https://www.cdc.gov/growthcharts/clinical_charts.htm. Accessed 15 Dec 2023

2. Mannarino S, Raso I, Garbin M, et al (2022) Cardiac dysfunction in Multisystem Inflammatory Syndrome in Children: An Italian single-center study. Ital J Pediatr 48:. https://doi.org/10.1186/S13052-021-01189-Z

3. Rostad BS, Shah JH, Rostad CA, et al (2021) Chest radiograph features of multisystem inflammatory syndrome in children (MIS-C) compared to pediatric COVID-19. Pediatr Radiol 51:231–238. https://doi.org/10.1007/S00247-020-04921-9

4. Giorno EPC, De Paulis M, Sameshima YT, et al (2020) Point-of-care lung ultrasound imaging in pediatric COVID-19. Ultrasound J 12:. https://doi.org/10.1186/S13089-020-00198-Z
